# Supplementary material for: Constructing asymmetric double-atomic sites for synergistic catalysis of electrochemical CO2 reduction
Source: Nat Commun. 2023 Oct 3;14:6164. doi: 10.1038/s41467-023-41863-w (PMC10547798; doi:10.1038/s41467-023-41863-w)
Supplement: Supplementary file 1 — Supplementary Information [file 41467_2023_41863_MOESM1_ESM.pdf]

1. Supplementary Figures

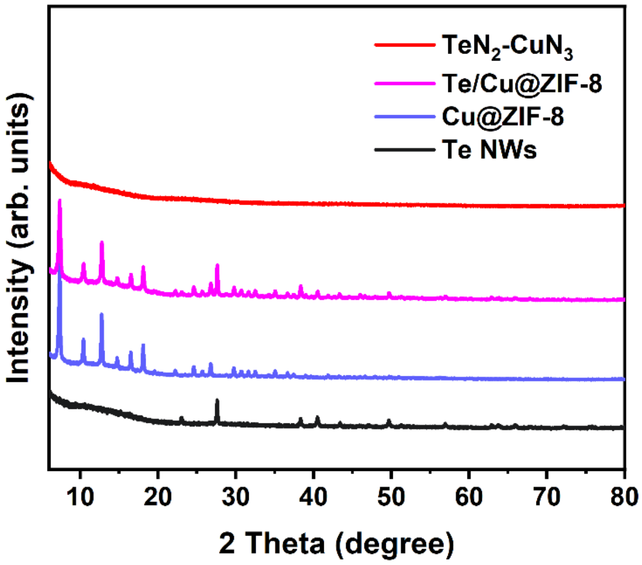

Supplementary Fig. 1 XRD patterns of TeN_2–CuN_3 DACs, core–sheath structured Te@ZIF-8 with Cu ions, ZIF-8 with Cu ions and Te nanowires.

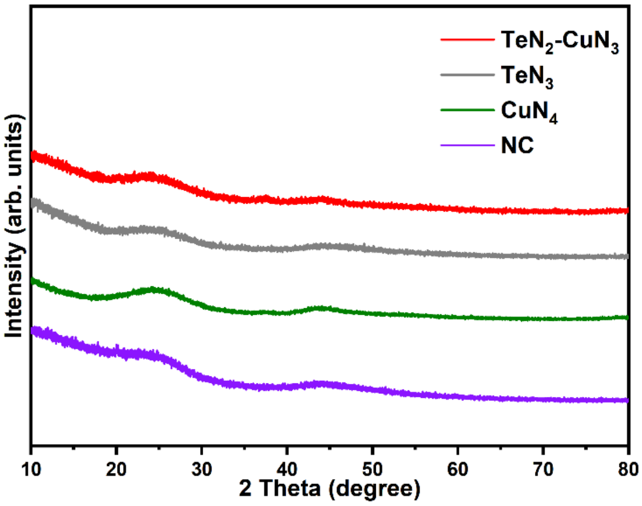

Supplementary Fig. 2 XRD patterns of TeN_2–CuN_3 DACs, TeN_3, CuN_4 and NC. No characteristic diffraction peaks of elemental Te or Cu were found, indicating no Te or Cu particles.

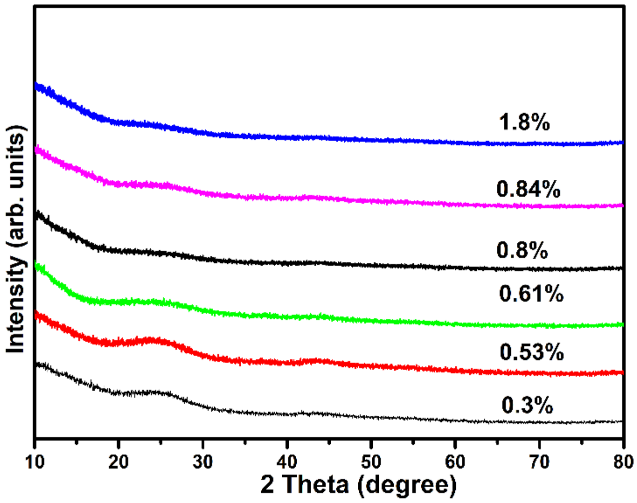

Supplementary Fig. 3 XRD patterns of catalysts with different Cu loadings. No characteristic diffraction peaks of elemental Cu were found, indicating no Cu particles.

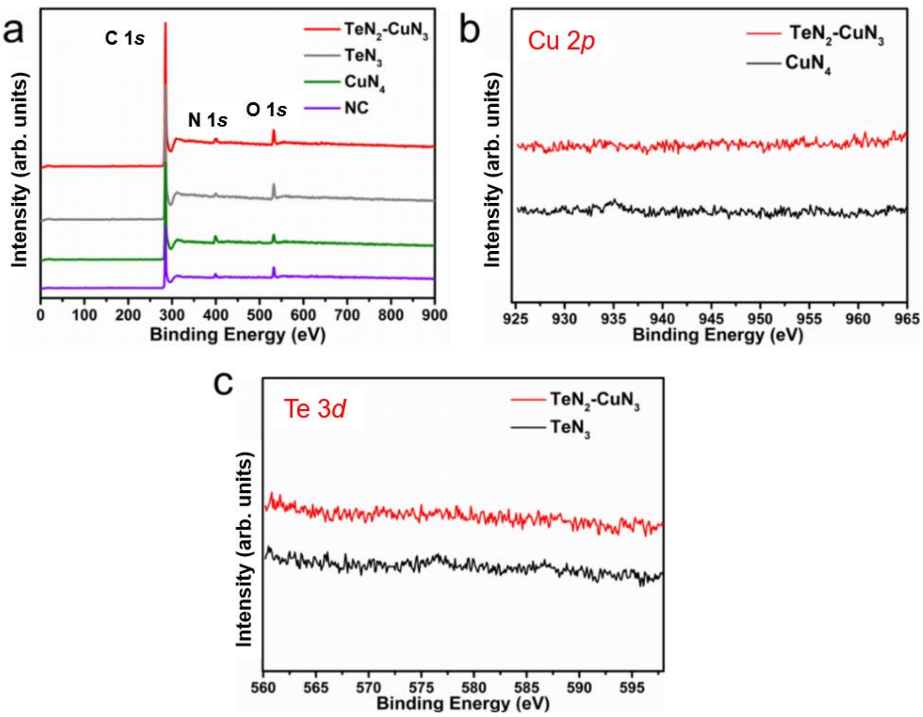

Supplementary Fig. 4 XPS spectra for different catalysts TeN_2–CuN_3 DAC, TeN_3, CuN_4 and NC. **a** XPS survey spectra. **b** High resolution XPS spectra of Cu 2*p* for TeN_2–CuN_3 and CuN_4. **c** High resolution XPS spectra of Te 3*d* for TeN_2–CuN_3 and TeN_3.

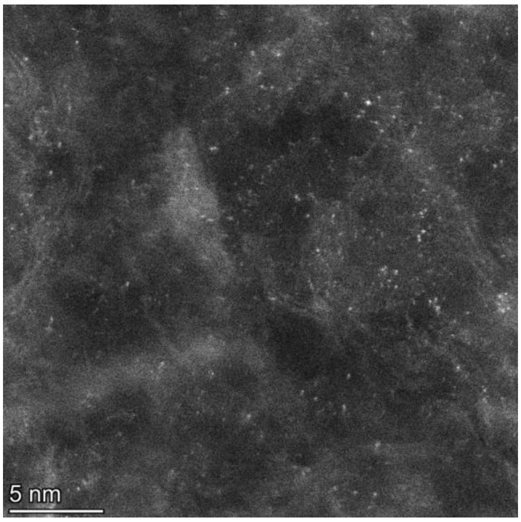

Supplementary Fig. 5 HAADF-STEM image of TeN_2–CuN_3 catalyst. The bright spots were identified as Te or Cu atoms. Most of the spots exist as pairs, indicative of double-atomic active sites.

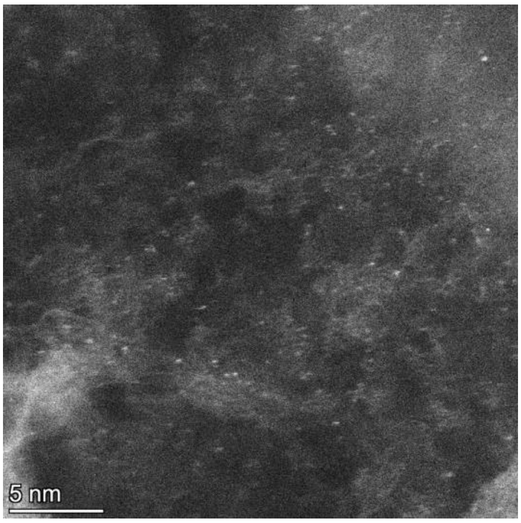

Supplementary Fig. 6 HAADF-STEM image of TeN_3 catalyst. The bright spots were identified as Te atoms. Most of the spots exist in isolation, indicative of single-atomic active sites.

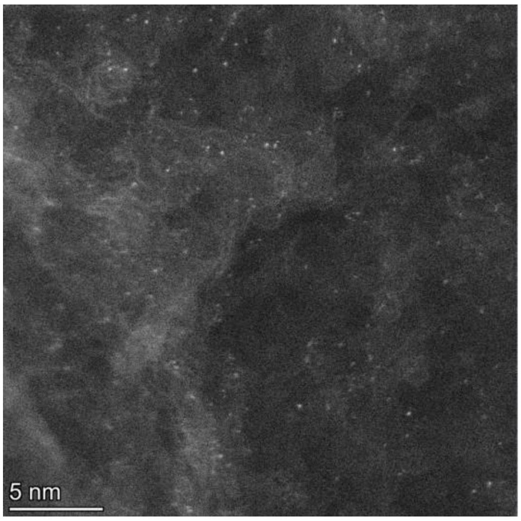

Supplementary Fig. 7 HAADF-STEM image of CuN_4 catalyst. The bright spots were identified as Cu atoms. Most of the spots exist in isolation, indicative of single-atomic active sites.

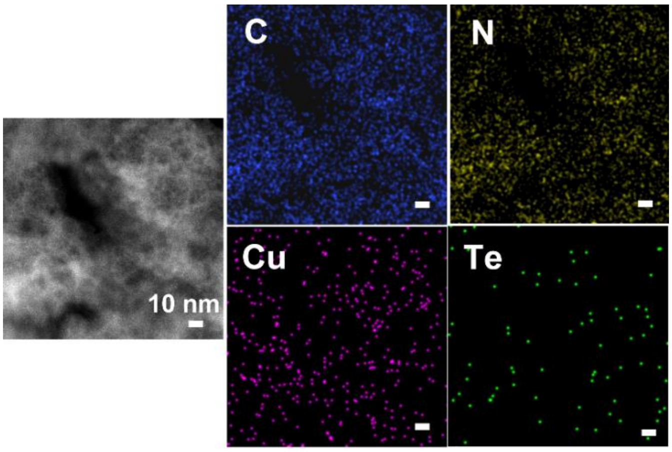

Supplementary Fig. 8 EDS mapping for different elements of TeN_2–CuN_3. Te and Cu elements were evenly dispersed on the N-doped carbon support.

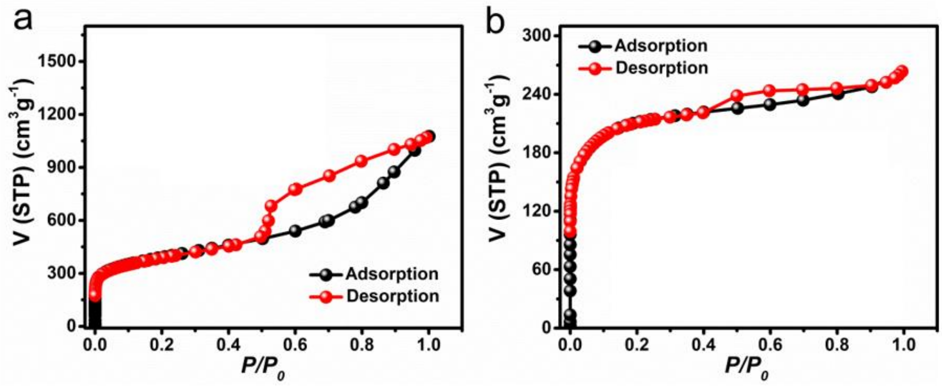

Supplementary Fig. 9 N_2 desorption curve of TeN_2–CuN_3 (a) and CuN_4 (b). The BET surface area of TeN_2–CuN_3 and CuN_4 BET are 1405.5 $m^2\ g^{-1}$ and 790.8 m^2g^{-1}, respectively.

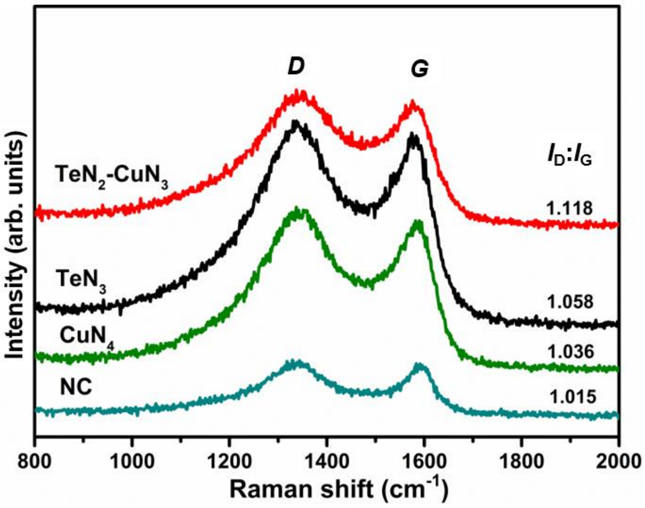

Supplementary Fig. 10 Raman spectra for TeN_2–CuN_3, TeN_3, CuN_4 and NC. The increased I_D/I_G ratio indicates that the formation of TeN_2–CuN_3 DAC induces more structural defects.

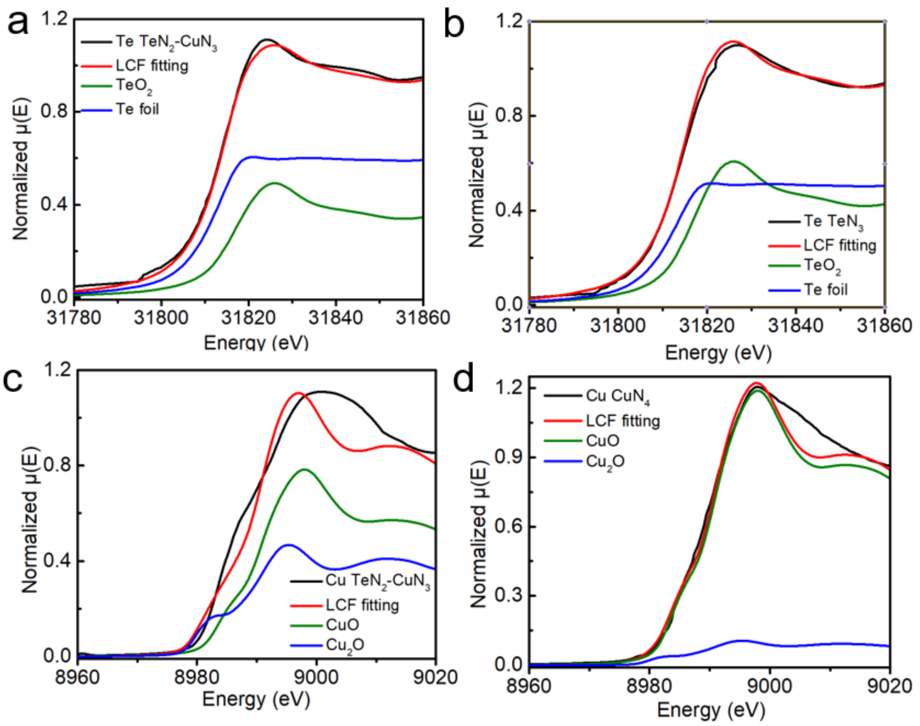

Supplementary Fig. 11 The linear combination fitting (LCF) of the Te of TeN_2–CuN_3(**a**) and TeN_3 (**b**), the corresponding Te foil and TeO_2 references. The LCF of Cu of TeN_2–CuN_3 (**c**) and CuN_4 (**d**), the corresponding CuO and Cu_2Oreferences.

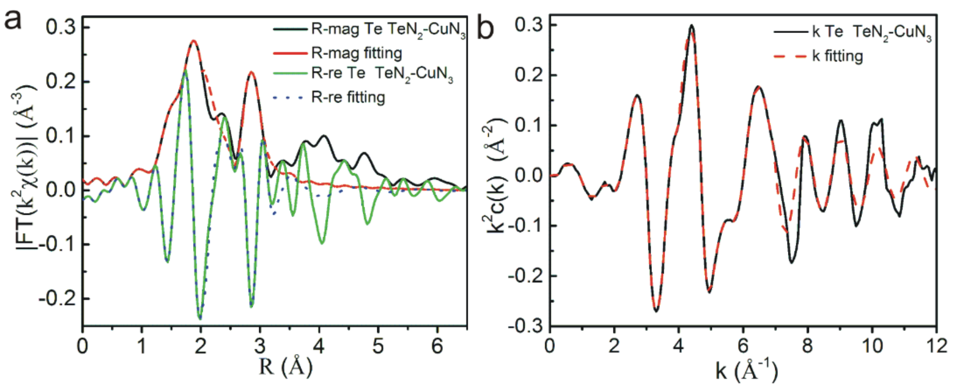

Supplementary Fig. 12 XAFS fitting results of Te for TeN_2–CuN_3. (**a**) χ(R) and (**b**) χ(k) space spectra fitting curve.

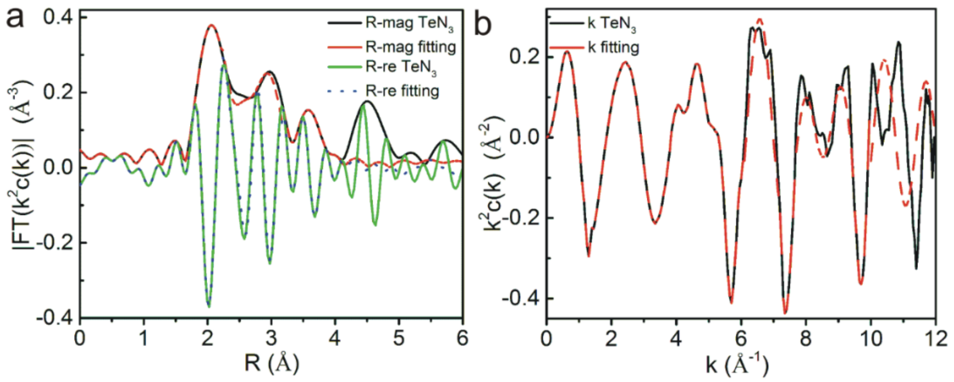

Supplementary Fig. 13 XAFS fitting results of Te for TeN_3. (a) $\chi(R)$ and **(b)** $\chi(k)$ space spectra fitting curve.

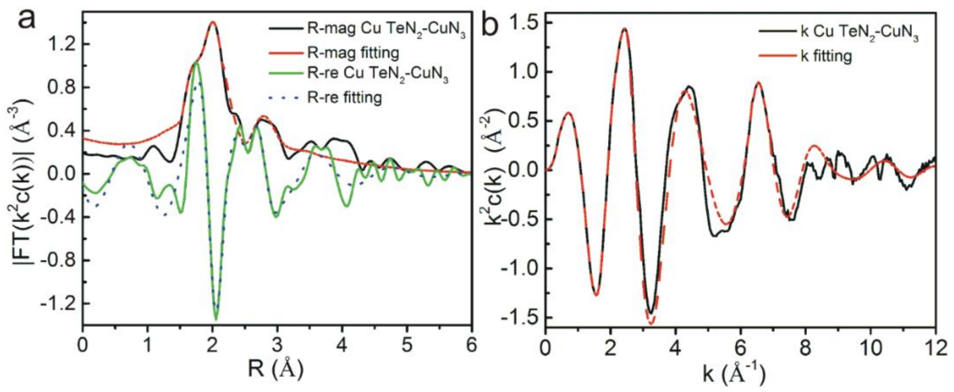

Supplementary Fig. 14 XAFS fitting results of Cu for TeN_2–CuN_3. (a) $\chi(R)$ and **(b)** $\chi(k)$ space spectra fitting curve of Cu for TeN_2–CuN_3.

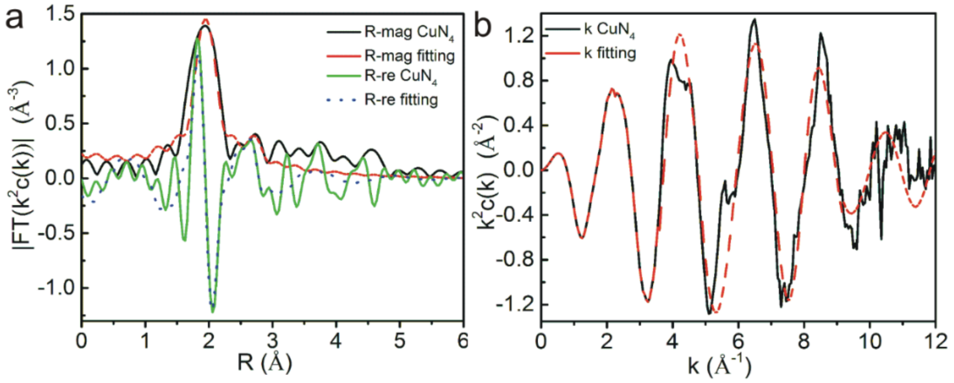

Supplementary Fig. 15 XAFS fitting results of Cu for CuN_4. (a) $\chi(R)$ and **(b)** $\chi(k)$ space spectra fitting curve.

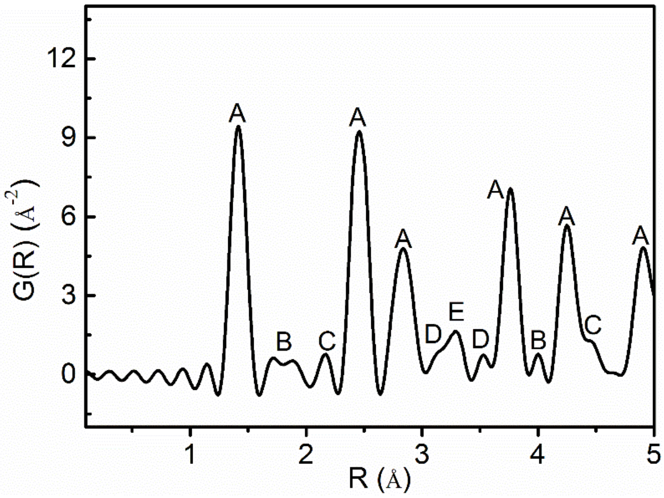

Supplementary Fig. 16 PDF data for TeN_2–CuN_3 DAC. The peaks labeled A correspond to C–N and C–C in N-doped C support; the peak at ~1.72 Å (labeled B), to Cu–N; the peak at ~2.15 Å (labeled C), to Te–N; the peak at ~1.88 Å, to Cu–N and Te–N (resulting from the asymmetric structure); the peaks at ~3.16 Å and 3.52 Å (labeled D), to Cu/Te–N–C; the peak at ~3.29 Å (labeled E), to Te–Cu.

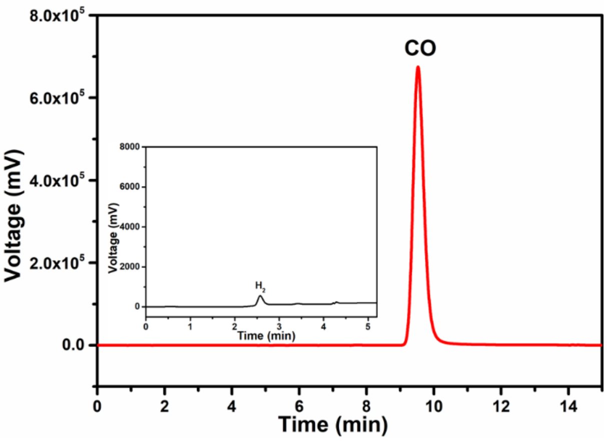

Supplementary Fig. 17 Gas chromatogram of TeN_2–CuN_3 catalyst for CO_2RR at -0.65 V (*vs*. RHE).

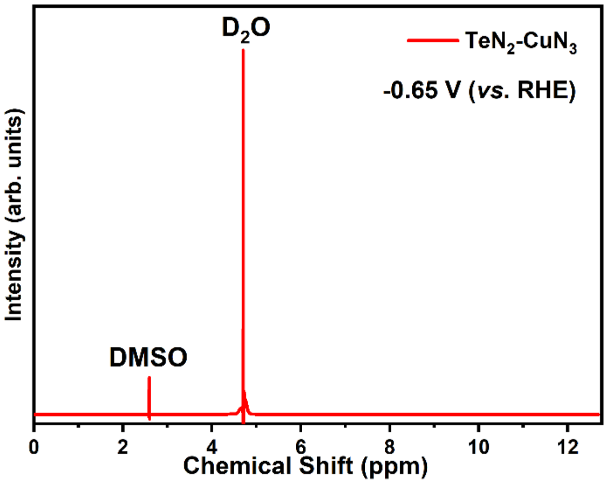

Supplementary Fig. 18 ^{1}H NMR spectra of TeN_2–CuN_3 catalyst for CO_2RR to produce liquid phase products at -0.65 V (*vs*. RHE).

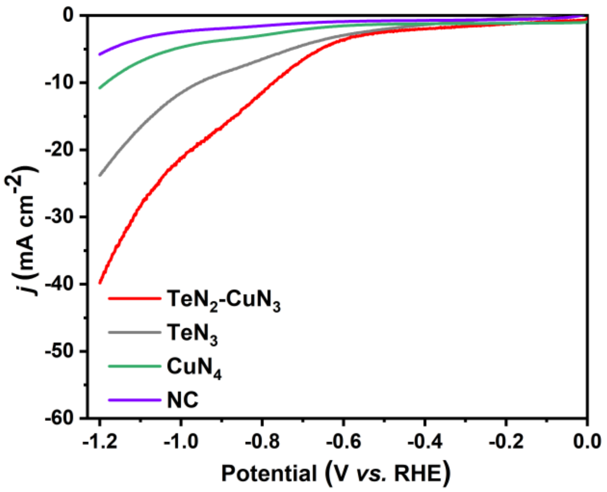

Supplementary Fig. 19 LSV curves of catalysts without *iR* correction of catalysts. (*R*, resistance: 9.0±0.6 ohm; electrode surface area: 0.25 cm^2). TeN_2–CuN_3 displays the highest total current density in the tested potential range.

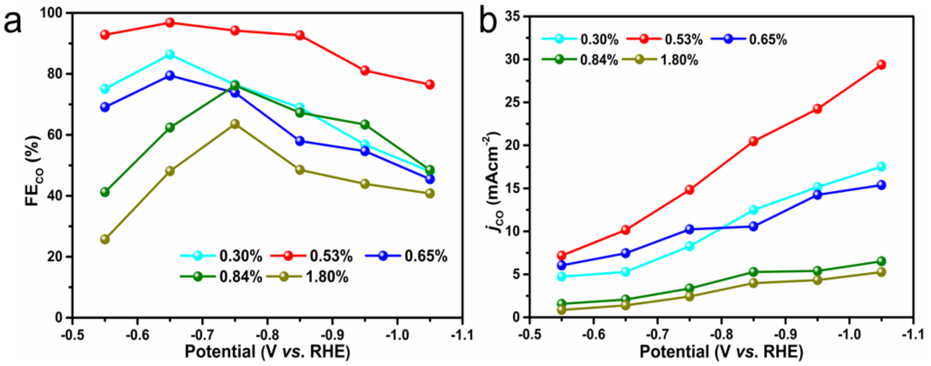

Supplementary Fig. 20 FE$_{CO}$ and j_{CO} for samples with different Cu loadings at different applied potentials. (**a**) FE$_{CO}$ and (**b**) j_{CO} for CO_2RR with TeN_2–CuN_3 catalysts with different Cu amount.

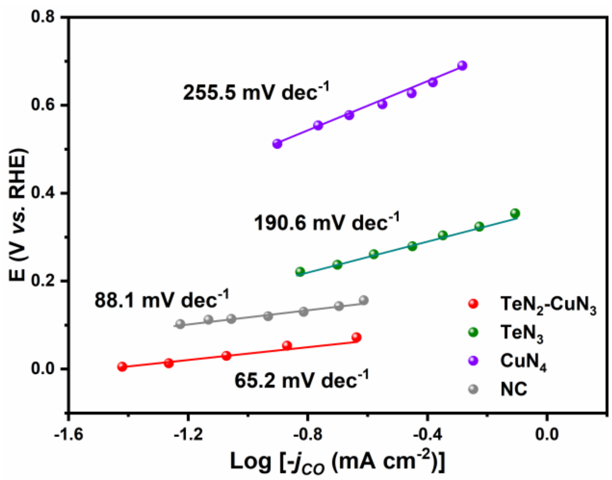

Supplementary Fig. 21 Tafel slopes for different samples. The TeN_2–CuN_3 displays the lowest Tafel slope, revealing a favorable reaction kinetics for CO generation.

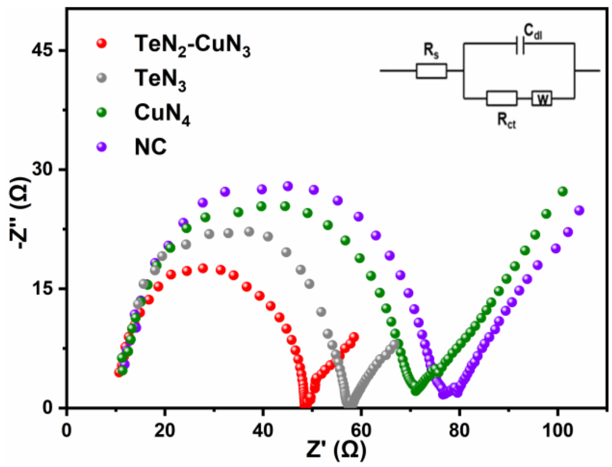

Supplementary Fig. 22 EIS spectra for different samples. The smallest semicircle diameter for TeN_2–CuN_3 indicates the fastest surface charge transfer, which also hints at a favorable reaction kinetics.

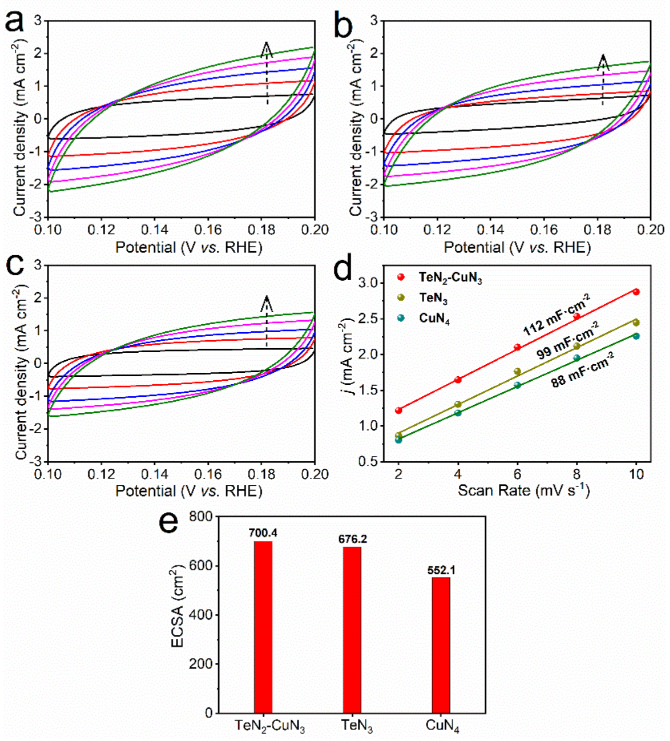

Supplementary Fig. 23 CV curves at various scan rates (from 2 to 10 mV s^{-1}) (**a-c**), and the corresponding C_{dl} (**d**) and ECSA values **(e)** for TeN_2–CuN_3, TeN_3, and CuN_4.

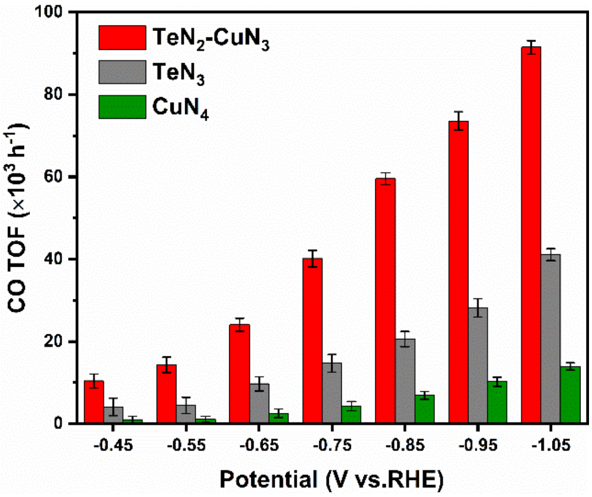

Supplementary Fig. 24 Calculated TOF for TeN_2–CuN_3, TeN_3 and CuN_4 at applied potentials. Error bars represent the standard deviation of three independent measurements.

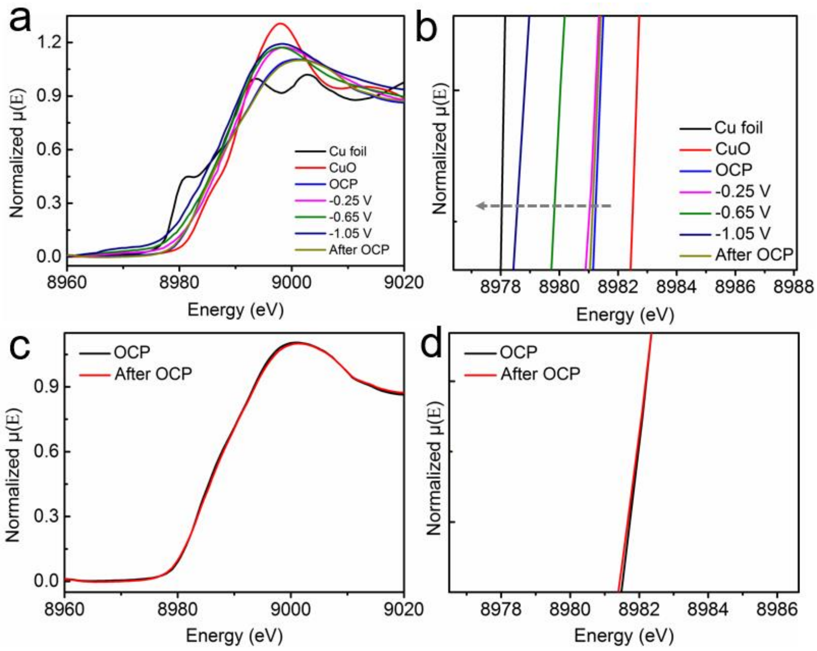

Supplementary Fig. 25 Operando XAFS spectra of TeN_2–CuN_3 DAC at Cu K-edge under CO_2RR in CO_2-saturated $KHCO_3$. **a** Potential dependence of operando Cu XAFS spectra. **b** Magnified absorption edge of XAFS region. Absorption edges under OCP condition (**c**) and after operando test (**d**).

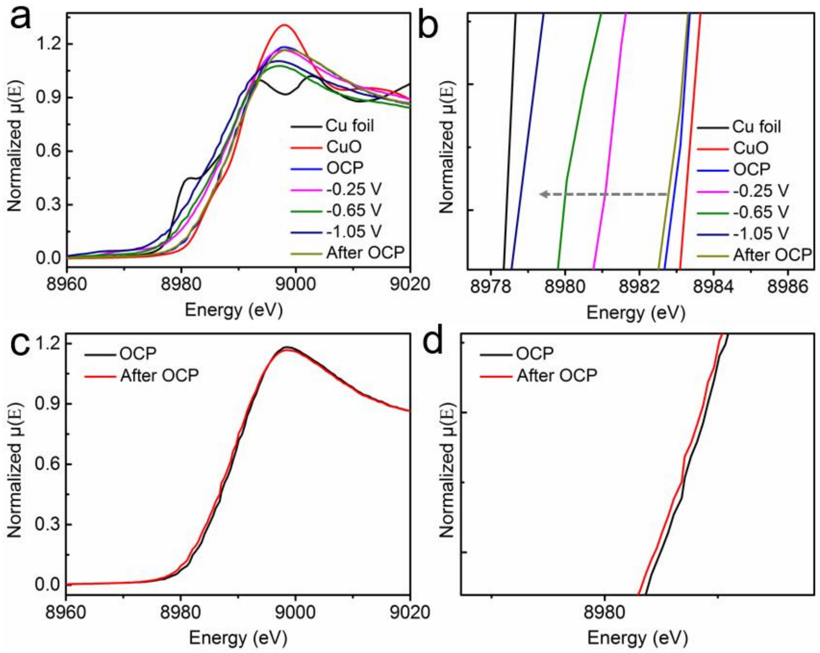

Supplementary Fig. 26 Operando XAFS spectra of CuN_4 SAC at Cu K-edge under CO_2RR in CO_2-saturated $KHCO_3$. **a** Potential dependence of operando Cu XAFS spectra**. b** Magnified absorption edge of XAFS region. Absorption edges under OCP condition (**c**) and after operando test (**d**).

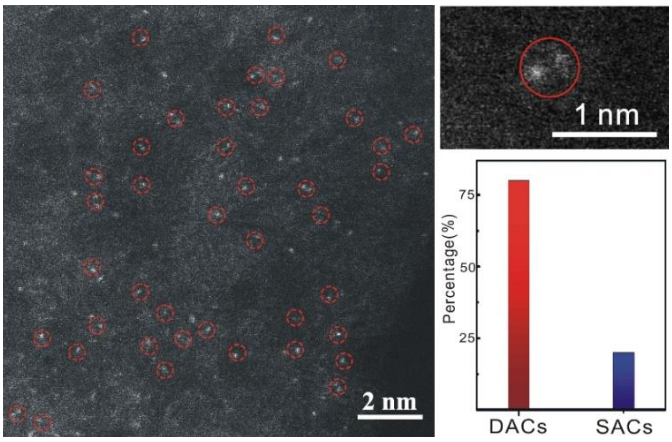

Supplementary Fig. 27 HAADF-STEM image of TeN_2–CuN_3 DAC after CO_2RR, with a typical Te–Cu dual-atom spot (top right) and statistical percentages for dual-atom and single-atom sites (bottom right).

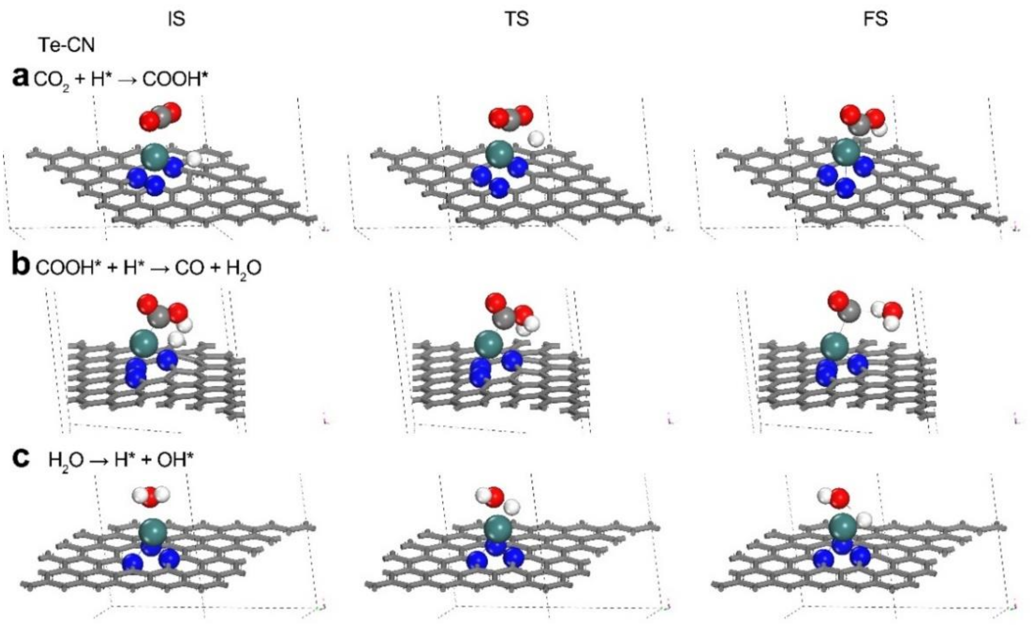

Supplementary Fig. 28 Structures initial (IS), transition (TS) and final (FS) states of (**a**) transition from CO_2 to COOH*, (**b**) transition from COOH* to CO and (**c**) transition from H_2O to H* and OH* over TeN_3 SAC catalyst.

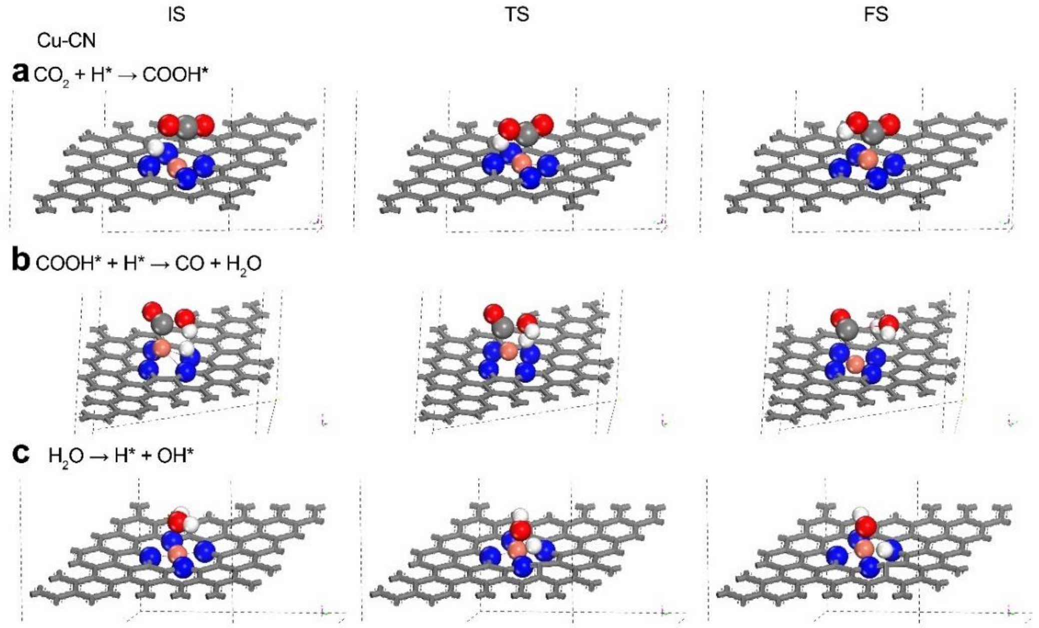

Supplementary Fig. 29 Structures initial (IS), transition (TS) and final (FS) states of (**a**) transition from CO_2 to COOH*, (**b**) transition from COOH* to CO and (**c**) transition from H_2O to H* and OH* over CuN_4 SAC catalyst.

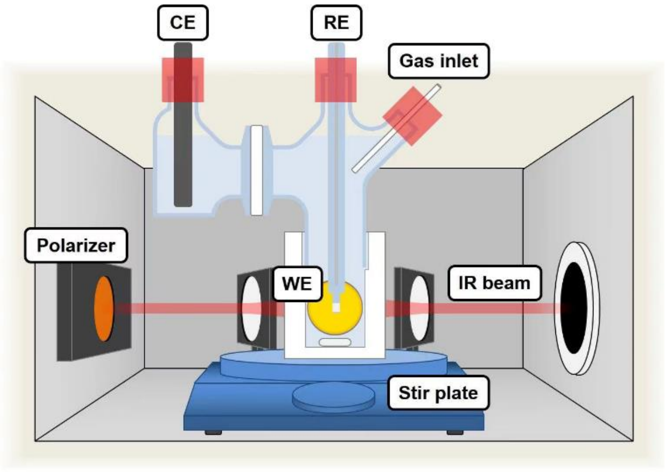

Supplementary Fig. 30 Schematic illustration of the spectroelectrochemical cell integrated with a stir plate that allows for stirring during the ATR-SEIRAS measurements. WE, RE and CE represent working electrode, reference electrode, and counter electrode, respectively.

2. Supplementary Tables

Supplementary Table1 The actual loadings (wt%) of Te and Cu forTeN_2–CuN_3, TeN_3 and CuN_4.

Samples	Te (wt%)	Cu (wt%)
TeN_2–CuN_3	0.56	0.53
TeN_3	0.58	0
CuN_4	0	0.55

Supplementary Table 2 The actual Cu loadings (wt%) with various Cu loadings.

Volume of $CuCl_2$ methanol	Cu (wt%)
10 uL	0.30
20 uL	0.53
40 uL	0.65
100 uL	0.84
200 uL	1.80

* The actual loading Te for samples are around 0.53wt%.

Supplementary Table 3 The exact valence state of Te and ratio of different samples quantified by LCF Method.

Samples	LCF fitting valance	Ratio of Te foil	Ratio of TeO_2
TeN_2–CuN_3	1.544	0.614	0.386
TeN_3	1.908	0.523	0.477

Supplementary Table 4 The exact valence state of Cu and ratio of different samples quantified by LCF Method.

Samples	LCF fitting valance	Ratio of CuO	Ratio of Cu_2O
TeN_2–CuN_3	1.599	0.599	0.401
CuN_4	1.909	0.909	0.091

Supplementary Table 5 Structural parameters extracted from the Te K-edge Te χ(R) space spectra fitting of TeN_2–CuN_3.

	Reduced Chi-square (χ_v^2)	R-factor (%)	amp/S_0^2	$N_{(Te\text{-}N\ path)}$	$R_{(Te\text{-}N\ path)}$ (Å)	$\sigma^2_{(Te\text{-}N\ path)}$ (10^{-3}Å^2)	ΔE_0 (eV)
Te TeN_2–CuN_3	664.60	0.0566	1.02+/-0.14	2	2.021±0.015	3.1+/-1.3	3.44+/-1.16
			amp/S_0^2	$N_{(Te\text{-}N\text{-}C\ path)}$	$R_{(Te\text{-}N\text{-}C\ path)}$ (Å)	$\sigma^2_{(Te\text{-}N\text{-}C\ path)}$ (10^{-3}Å^2)	ΔE_0 (eV)
			0.86+/-0.11	4	3.091±0.032	4.3+/-1.8	3.37+/-1.89

Supplementary Table 6 Structural parameters extracted from the Te K-edge Te χ(R) space spectra fitting of TeN_3.

	Reduced Chi-square (χ_v^2)	R-factor (%)	amp/S_0^2	$N_{(Te\text{-}N\ path)}$	$R_{(Te\text{-}N\ path)}$ (Å)	$\sigma^2_{(Te\text{-}N\ path)}$ (10^{-3}Å^2)	ΔE_0 (eV)
TeN_3	624.39	0.0424	1.05+/-0.13	3	1.967 ± 0.062	3.3+/-1.7	3.89+/-1.41
			amp/S_0^2	$N_{(Te\text{-}N\text{-}C\ path)}$	$R_{(Te\text{-}C\ path)}$ (Å)	$\sigma^2_{(Te\text{-}C\ path)}$ (10^{-3}Å^2)	ΔE_0 (eV)
			0.84+/-0.13	6	2.731±0.087	4.4+/-2.2	4.03+/-1.89
			amp/S_0^2	$N_{(Te\text{-}N\text{-}C\ path)}$	$R_{(Te\text{-}C\ path)}$ (Å)	$\sigma^2_{(Te\text{-}C\ path)}$ (10^{-3}Å^2)	ΔE_0 (eV)
			0.84+/-0.13	3	3.066±0.098	4.4+/-2.2	4.03+/-1.89

Supplementary Table 7 Structural parameters extracted from the Cu K-edge Cu χ(R) space spectra fitting of TeN_2–CuN_3.

Cu TeN_2–CuN_3	Reduced Chi-square (χ_v^2)	R-factor (%)	amp/S_0^2	$N_{(Cu\text{-}N\ path)}$	$R_{(Cu\text{-}N\ path)}$ (Å)	$\sigma^2_{(Cu\text{-}N\ path)}$ (10^{-3}Å^2)	ΔE_0 (eV)
	499.11	0.0518	0.83+/-0.12	3	1.991 ± 0.086	2.4+/-1.0	2.04+/-1.22
			amp/S_0^2	$N_{(Cu\text{-}N\text{-}C\ path)}$	$R_{(Cu\text{-}N\text{-}C\ path)}$ (Å)	$\sigma^2_{(Cu\text{-}N\text{-}C\ path)}$ (10^{-3}Å^2)	ΔE_0 (eV)
			0.84+/-0.11	5	2.811±0.076	3.6+/-1.2	2.95+/-1.36

Supplementary Table 8 Structural parameters extracted from the Cu K-edge Cu χ(R) space spectra fitting of CuN_4.

CuN_4	Reduced Chi-square (χ_v^2)	R-factor (%)	amp/S_0^2	$N_{(Cu\text{-}N\ path)}$	$R_{(Cu\text{-}N\ path)}$ (Å)	$\sigma^2_{(Cu\text{-}N\ path)}$ (10^{-3}Å^2)	ΔE_0 (eV)
	661.60	0.0478	0.83+/-0.17	4	1.948 ± 0.077	3.4+/-1.5	2.46+/-1.29
			amp/S_0^2	$N_{(Cu\text{-}N\text{-}C\ path)}$	$R_{(Cu\text{-}C\ path)}$ (Å)	$\sigma^2_{(Cu\text{-}C\ path)}$ (10^{-3}Å^2)	ΔE_0 (eV)
			0.80+/-0.12	4	2.789± 0.082	4.1+/-1.9	4.54+/-1.54

Supplementary Table 9 Table peaks assignment to the atomic pair distance for TeN_2–CuN_3 catalyst.

**Label**	**Atomic pair**	**Atomic pair distances (Å)**
A	C–C, C–N	1.44, 2.44, 2.83, 3.76, 4.25, 4.90
B	Cu–N	1.73, 1.88, 4.01
C	Te–N	1.88, 2.15, 4.45
D	Cu–N–C, Te–N–C	3.16, 3.52
E	Te–Cu	3.27
